# New 2-Acetyl-3-aminophenyl-1,4-naphthoquinones: Synthesis and *In Vitro* Antiproliferative Activities on Breast and Prostate Human Cancer Cells

**DOI:** 10.1155/2020/8939716

**Published:** 2020-09-26

**Authors:** David Ríos, Jaime A. Valderrama, Miriam Cautin, Milko Tapia, Felipe Salas, Angélica Guerrero-Castilla, Giulio G. Muccioli, Pedro Buc Calderón, Julio Benites

**Affiliations:** ^1^Química y Farmacia, Facultad de Ciencias de la Salud, Universidad Arturo Prat, Casilla 121, Iquique 1100000, Chile; ^2^Bioanalysis and Pharmacology of Bioactive Lipids (BPBL), Louvain Drug Research Institute, Université Catholique de Louvain, 72 Avenue E. Mounier, BPBL 7201, 1200 Brussels, Belgium; ^3^Research Group in Metabolism and Nutrition, Louvain Drug Research Institute, Université Catholique de Louvain, 73 Avenue E. Mounier, 1200 Brussels, Belgium

## Abstract

The reaction of 2-acyl-1,4-naphthoquinones with *N*,*N*-dimethylaniline and 2,5-dimethoxyaniline, promoted by catalytic amounts of CeCl_3_·7H_2_O under “open-flask” conditions, produced a variety of 2-acyl-3-aminophenyl-1,4-naphthoquinones structurally related to the cytotoxic 2-acetyl-3-phenyl-1,4-naphthoquinone, an inhibitor of the heat shock chaperone protein Hsp90. The members of the 2-acyl-3-aminophenyl-1,4-naphthoquinone series were isolated in good yields (63-98%). The cyclic voltammograms of the 2-acyl-3-aminophenyl-1,4-naphthoquinone exhibit two one-electron reduction waves to the corresponding radical-anion and dianion and two quasireversible oxidation peaks. The first and second half-wave potential values (*E*_1/2_) of the members of the series are sensitive to the push-pull electronic effects of the substituents in the naphthoquinone scaffold. Furthermore, the *in vitro* antiproliferative properties of these new quinones were evaluated on two human cancer cells DU-145 (prostate) and MCF-7 (mammary) and a nontumorigenic HEK-293 (kidney) cell line, using the MTT colorimetric method. Two members, within the series, exhibited interesting cytotoxic activities on human prostate and mammary cancer cells.

## 1. Introduction

The electroactive naphthoquinone core is a common structural constituent of a variety of biochemical systems involved in the human defense system [1]. Several biological active quinones as ubiquinone and vitamin K are known to be electron transporters and are essential for many enzymatic processes. They can act as anti- or prooxidants depending on the nature of the media: this chemical versatility gives them an important role in different biochemical processes that are essential to living organisms [2]. The oxidation state of naphthoquinones allows them to act by different mechanisms, such as free radical scavengers, metal ion chelators, and also enzyme inhibitors for free radical production [3, 4]. This imbalance between the formation and removal of ROS (reactive oxygen species) causes damage to the cells at nucleic acids, proteins, and membrane lipids associated with ageing, carcinogenesis, cardiovascular, and coronary diseases [5]. In this context, the naturally occurring 2-hydroxy-1,4-naphthoquinone (lawsone) exhibits a number of interesting biological activities, such as antioxidant [6], antibacterial, antifungal [7], anti-inflammatory, antipyretic, analgesic [8], and anticancer cytotoxic [9]. Therefore, the quinones could play an important role in inhibiting or delaying oxidative stress that arises from an imbalance between free radical production and antioxidant and repair defenses [10].

**Figure 1:**
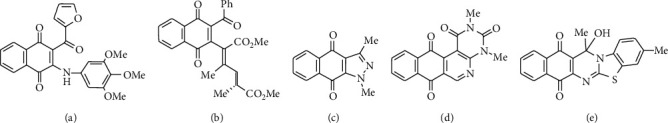
Examples of naphthoquinone-containing compounds prepared from acylquinones.

Acylated 1,4-quinones such as 2-acyl-1,4-benzo- and 2-acyl-1,4-naphthoquinones are a valuable building block of several natural [1, 11–13] and synthetic compounds endowed with a wide range of biological activities [14–19]. Acylquinones are highly reactive towards nucleophiles due to the confluence of electrophilic centers at the quinone nucleus and the 2-carbonyl substituents. The relatively close location of these electrophilic centers in the acylquinone scaffold enables reactions with diverse mono- and binucleophiles such as arylamines [17], azaenamines [18], enaminones [19], and 2-aminobenzothiazoles [20] to give a diversity of substances shown in Figure 1 such as 2,3-disubstituted 1,4-naphthoquinones (a, b) and heterocyclic fused 1,4-naphthoquinones (c–e).

**Figure 2:**
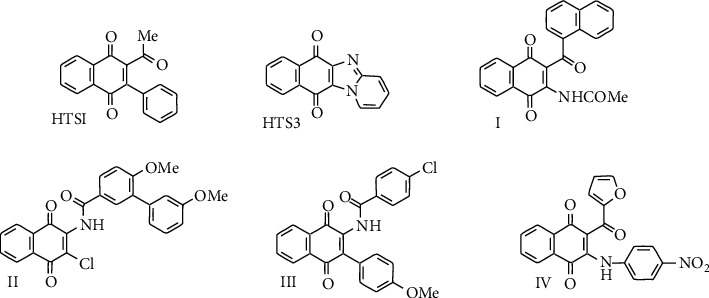
Structure of HTS1/HTS3 and quinone-analogue inhibitors of Hsp90.

High-throughput screening of a library of structurally diverse compounds has identified, among 120 active members, the 1,4-naphthoquinone derivatives, named HTS1 and HTS3, as a new class of inhibitors of the heat shock chaperone protein Hsp90 (Figure 2). The protein Hsp90, which ensures protein homeostasis in the presence and in the absence of cellular stress, is unique because most of its client proteins are conformationally labile signal transducers that play a crucial role in cell growth control, survival, and development processes [11]. Hsp90 represents 1–2% of the total protein cellular content, but its expression is enhanced by 2–10-fold in cancer cells [12], thus making it an attractive goal for the development of Hsp90 inhibitors [1, 13–15] and, consequently, a relevant target in cancer therapy [21]. Both HTS1 and HTS3 have been shown antiproliferative activity against human cancer mammary cells. For instance, HTS1 showed 4-fold greater antiproliferative activity against MCF-7 cells (an estrogen-dependent breast cancer cell line) compared to SKBr3 cells (an Her2-overexpressing breast cancer cell line), indicating that this scaffold may provide a useful probe to study estrogen-dependent cancers. On the other hand, HTS3 shows equal activity against both mammary cancer cells. In addition, the members I-III induced the degradation of oncogenic Hsp90 client proteins, a hallmark of Hsp90 inhibition [22].

Recently, we have reported the synthesis of a variety of 2-acyl-3-phenylamino-1,4-naphthoquinones (i.e., compound IV in Figure 2) prepared by oxidative amination of 2-acylnaphthoquinones with phenylamines. The members of this naphthoquinone series were designed as potential inhibitors of Hsp90 chaperoning function due to their close structural similarity to that of HTS1. The *in vitro* screening of the series demonstrated cytotoxic activity on cancer cells [23], and the congener IV (Figure 2) exhibited action as an inhibitor of Hsp90 chaperoning function [19].

Older studies reported by Pardo et al. [24] demonstrated that the reaction of 2-acetylnaphthohydroquinone with phenylamines, under oxidant conditions, takes place in a complex manner. Indeed, it yields four types of products depending upon the structure of the arylamine and the medium solvent: amination (C-N bond formation), arylation (C-C bond formation), arylation-cyclisation (C-C and C=N bond formations), and arylation-amination (C-C and N-C bond formations) [24, 25]. These reactions occur between the “nascent” 2-acetyl-1,4-naphthoquinone, *in situ* generated by oxidation of acetylnaphthohydroquinone with sodium periodate, and the arylamines. Recent studies performed in our laboratory show that the reaction of diverse 2-acylnaphthoquinones with 3,4,5-trimethoxyaniline in methanol yields amination products (C-N bond formation) together with two types of arylation-cyclisation products (C-C and two alternative N=C or N-C bond formations). We have not detected arylation products in these assays probably due to the fact that they undergo fast cyclisation reactions to produce their respective arylation-cyclisation products [26].

**Scheme 1:**
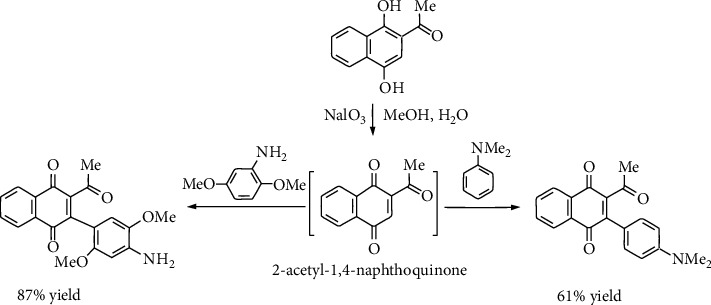
Formation of 2-acetyl-3-aminophenyl-1,4-naphthoquinones. Adapted from [24].

Pardo et al. [24] reported the synthesis of arylation products, named as 2-acetyl-3-aminophenyl-1,4-naphthoquinones (Scheme 1), which are closely related to HTS1 (2-acetyl-3-phenyl-1,4-naphthoquinone) shown in Figure 2. Inspired by such structural similarities, we wanted to explore the acylquinone scope of this arylation reaction to the synthesis of 2-acyl-3-aminophenylnaphthoquinones from a representative number of acylnaphthoquinones, *N*,*N*-dimethylaniline and 2,5-dimethoxyaniline.

In this paper, we report flexible access to 2-acyl-3-aminophenyl-1,4-naphthoquinones prepared from 2-acylnaphthohydroquinones, *N*,*N*-dimethylaniline and 2,5-dimethoxyaniline. The half-wave potentials of such new HTS1 analogues were calculated, and they were evaluated for their *in vitro* antiproliferative activities on two human-derived cancer cell lines DU-145 (prostate) and MCF-7 (breast) and nontumorigenic HEK-293 (kidney) cells using as endpoint the MTT colorimetric method.

## 2. Materials and Methods

### 2.1. General Information

All the solvents and reagents were purchased from different companies, such as Aldrich (St. Louis, MO, USA) and Merck (Darmstadt, Germany), and were used as supplied. Melting points (mp) were determined on a Stuart Scientific SMP3 (Staffordshire, UK) apparatus and are uncorrected. The record of IR, ^1^H- and ^13^C-NMR spectra, and chromatography procedures were done according to methods reported by Benites et al. [27]. Proton nuclear magnetic resonance (^1^H NMR) spectra were measured at 300 MHz in a Bruker Ultrashield-300 spectrometer. Data for the ^1^H NMR spectra are reported as follows: s = singlet, br s = broad singlet, d = doublet, and t = triplet, and the coupling constants (*J*) are in Hz. Carbon-13 nuclear magnetic resonance (^13^C NMR) spectra were measured at 75 MHz in a Bruker Ultrashield-300 spectrometer. Bidimensional NMR techniques and distortion-less enhancement by polarization transfer (DEPT) were used for the signal assignment. Chemical shifts are expressed in ppm downfield relative to tetramethylsilane, and the coupling constants (*J*) are reported in Hertz. The high-resolution mass spectrometry (HRMS) data for all final compounds were obtained using a LTQ-Orbitrap mass spectrometer (Thermo-Fisher Scientific, Waltham, MA, USA) with the analysis performed using an atmospheric-pressure chemical ionization (APCI) source, operated in a positive mode. The acylnaphthohydroquinones (2-8) were prepared according to a previously reported procedure [28].

### 2.2. Chemistry

#### 2.2.1. Preparation of 2-Acyl-3-aminophenylnaphthoquinones (16-28): General Procedure

Suspensions of the acylnaphthohydroquinones 2–8 (1.0 mmol), Ag_2_O (2.0 equiv.), and MgSO_4_ anhydrous (300 mg) in dichloromethane (30 mL) were left with stirring for 30 min at room temperature (rt). The mixtures were filtered, the solids were washed with dichloromethane (3 × 15 mL), and the filtrates containing the respective 2-acyl-1,4-naphthoquinones were evaporated under reduced pressure. The residues were dissolved in methanol (15 mL), the phenylamines (2 equiv.) and CeCl_3_·7H_2_O (5% mmol) were added to the solutions, and the mixtures were left with stirring at rt according to the times collected in Table 1. The solvents were removed under reduced pressure, and the residues were column-chromatographed over silica gel (petroleum ether/EtOAc) to yield the corresponding pure 2-acyl-3-aminophenylnaphthoquinones 16-28.

### 2.3. Biological Assays

#### 2.3.1. Cell Lines and Cell Cultures

Human cancer cell lines MCF-7 (mammary) and DU-145 (prostate) and nontumor HEK-293 cells were obtained from the American Type Culture Collection (ATCC, Manassas, VA, USA). The cultures were maintained at a density of 1 × 10^5^ cells/mL, and the medium was changed at 48 to 72 h intervals. They were cultured in high-glucose Dulbecco's modified Eagle medium (Gibco, Grand Island, NY, USA) supplemented with 10% fetal calf serum, penicillin (100 U/mL), and streptomycin (100 *μ*g/mL). All cultures were kept at 37°C in 95% air/5% CO_2_ at 100% humidity. Phosphate-buffered saline (PBS) was purchased from Gibco. Cells were incubated at the indicated times at 37°C, with or without quinones at various concentrations.

#### 2.3.2. Cytotoxic Assays

The cytotoxicity of the quinones was assessed by the MTT (3-(4,5-dimethylthiazol-2-yl)-2,5-diphenyltetrazolium bromide) reduction assays [29], according to Valderrama et al. [30]. Briefly, adherent cells were detached by using trypsin/EDTA solution. The culture medium was removed, and cells were washed with a free Ca-Mg salt solution to remove all traces of serum. After removing salt solution, trypsin/EDTA solution was added to completely cover the monolayer of cells for 2-3 min at 37°C. When the trypsinization process was completed, trypsin/EDTA was removed by aspiration and cells were resuspended, diluted in fresh medium, and seeded for 24 h into 96-well plates at a density of 10,000 cells/well. Then, they were further incubated for 48 h, with or without the quinone derivatives. Doxorubicin was used as the standard chemotherapeutic agent (positive control) in a dose range of 0.01 to 10 *μ*M. Cells were then washed twice with warm PBS, and they were further incubated with MTT (0.5 mg/mL) for 2 hours at 37°C. Blue formazan crystals were solubilized by adding 100 *μ*L DMSO/well, and the optical density of the colored solutions was subsequently read at 550 nm. Results are expressed as a percentage of MTT reduction, compared to untreated control conditions. The IC_50_ values were calculated using the GraphPad Prism software (San Diego, CA, USA).

## 3. Results and Discussion

### 3.1. Chemistry

**Scheme 2:**
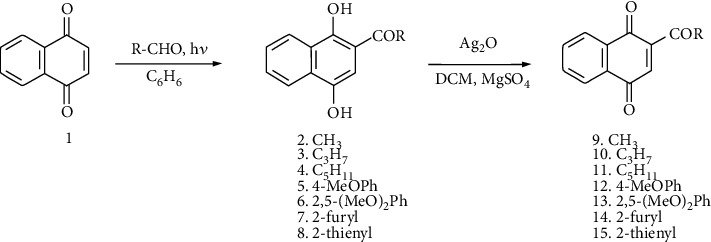
Preparation of 2-acylnaphthoquinones 9-15 from 1 and diverse aldehydes.

The 2-acylnaphthoquinones 9-15 selected for the study were prepared from the 2-acylnaphthohydroquinones 2-8 according to Scheme 2. The hydroquinone precursors 2-8 were synthesized by solar photo-Friedel-Crafts acylation of 1,4-naphthoquinone 1 with the following aldehydes: acetaldehyde, butyraldehyde, hexanal, 4-methoxybenzaldehyde, 2,5-dimethoxybenzaldehyde, 2-furancarbaldehyde, and 2-thiophencarbaldehyde, according to our previously reported procedure [28]. Access to the 2-acyl-1,4-naphthoquinones 9-15 was accomplished by oxidation of 2-acylnaphthohydroquinones 2-8 with silver (I) oxide in dichloromethane in the presence of dry magnesium sulphate [19]. The resulting acylnaphthoquinones isolated from the mixture reactions were dissolved in methanol and immediately reacted with the *N*,*N*-dimethylaniline and 2,5-dimethoxyaniline (Supplementary Materials (available here)).

Among the members of the acylnaphthoquinone series, compound 14 was selected to get preliminary insights into its reactivity to undergo arylation reaction with *N*,*N*-dimethylaniline. In a preliminary assay, acylnaphthoquinone 14, prepared from acylnaphthohydroquinone 7, was reacted with *N*,*N*-dimethylaniline in methanol at rt. The reaction carried out under open-flask conditions to favor aerobic oxidation reactions takes place slowly to give, after 72 hours, the arylation product 20 in 73% yield, referred to precursor 7. Furthermore, it was observed that the reaction of 14 with *N*,*N*-dimethylaniline in refluxing ethanol takes place relatively faster (33 hours) than in methanol, but compound 20 is produced in moderate yield (51%).

The validity of the arylation mechanism of acylquinone 9 with arylamines proposed by Pardo et al. [24] was assumed to proceed through a sequence of a Michael addition reaction followed by aerobic oxidation of the adduct intermediate. Therefore, we envisaged to improve the arylation reaction of 14 (Table 1) with *N*,*N*-dimethylaniline by using a green Lewis acid catalyst such as CeCl_3_·7H_2_O [31] or IBr_3_ [32]. Successful results on the use of the Ce(III) catalyst in the oxidative amination reaction of quinones with arylamines have been reported in literature [32–38]. These acid catalysts probably increase the electrophilic character of the enone system of the quinones, *via* coordination with the oxygen atom of the carbonyl group, thus promoting the Michael-type addition [35, 39, 40].

We examined the reactions of 14 with *N*,*N*-dimethylaniline in the presence of catalytic amounts of CeCl_3_·7H_2_O and IBr_3_ (5%) in methanol, under open-flask conditions at room temperature. Both reactions occurred faster (28 h) than in the absence of these Lewis acids (72 h), and the arylation product 20 (Table 1) was isolated in 98 and 78% yield, respectively. The successful results obtained in the assay employing Ce(III) led us to evaluate the scope of the arylation reaction of the remaining members of the series with the selected arylamines, under the above optimized conditions. The results of the assays are summarized in Table 1.

The structures of compounds 16–28 were established by ^1^H- and ^13^C-nuclear magnetic resonance (NMR), bidimensional nuclear magnetic resonance (2D-NMR), and high-resolution mass spectrometry (HRMS).

The data in Table 1 show that the arylation reaction of 2-acylnaphthoquinones 9-15 with the arylamines, catalyzed with Ce(III), yields the corresponding 2-acetyl-3-aminoaryl-1,4-naphthoquinones with good to excellent yields ranging from 63 to 98%, except in the case of product 17 (33%). Comparison of the reaction time formation of compounds 16-28 reveals two facts: the first one was linked to the lower reactivity of *N*,*N*-dimethylaniline compared to 2,5-dimethoxyaniline and secondly the strong influence of the stereoelectronic nature of the acyl substituents on the nucleophilic attack of the arylamines. It has to be mentioned that no attempts were made in order to decrease the reaction time formation of the arylation products. Based on our recent results [41] and that reported by Liu and Ji [42], on the arylamination of quinones, we will attempt, in future researches, the use of ultrasound to accelerate the Ce(III)-promoted arylation reaction of acylnaphthoquinones with arenes in order to expand the 2-acetyl-3-aminoaryl-1,4-naphthoquinone series for further biological studies.

**Scheme 3:**
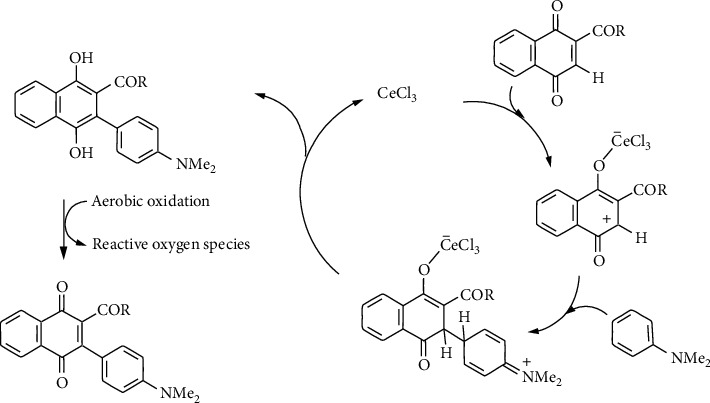
Proposed reaction mechanism for the Ce(III)-promoted arylation reaction of acylnaphtoquinones with *N*,*N*-dimethylaniline.

The acid-induced formation of the arylation compounds 16-28 from acylquinones 9-15 and their reaction with *N*,*N*-dimethylaniline are proposed to occur according to the mechanism of reaction depicted in Scheme 3. This approach is based on the reaction mechanism for the Ce(III)-promoted phenylamination reaction of 1,4-naphthoquinone with 2-fluoro- and 2-methoxyanilines reported by Leyva et al. [35]. Initially, a selective conjugate Michael-type addition of the arylamine across the enone system C_3_=C_2_-C_1_=O seems plausible. This reaction, which involves the high electrophilic C-3 of the acylquinones, provides the respective C-C Michael adduct intermediates. Further enolization of these species, followed by aerobic oxidation, gives compounds 16-21. It should be noted that the electrophilicity of the C-3 in these acylquinones is mainly due to the electron-withdrawing effects of the acyl substituents attached to the 2-position. The coordination of Ce(III) to the oxygen atom of the enone system should contribute to increasing the electrophilicity of the C-3.

**Figure 3:**
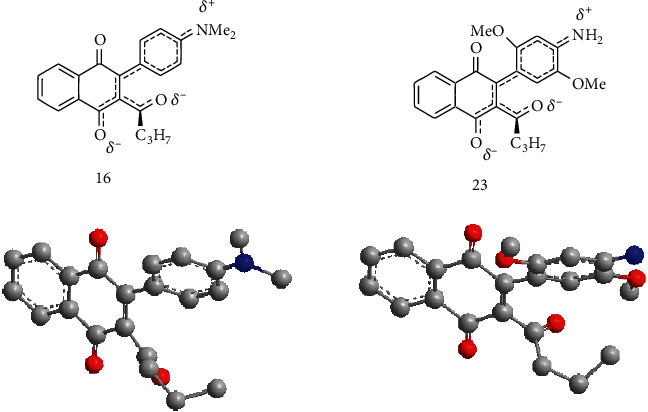
Hybrid and 3-D optimized structure of compounds 16 and 23.

An interesting feature of the synthetized arylation products is their strong purple color of the chromophores that, according to Pardo et al. [24], is due to the strong donor-acceptor interactions between the quinoid and the electron-rich nitrogen substituents.  Figure 3 shows the hybrid structures of compounds 16 and 23 where such interactions are clearly observable. Inspection of minimal energy conformation of compounds 16 and 23 performed by MM2 calculation (ChemBio3D 11.0, PerkinElmer, MA, USA) shows a noncoplanar orientation between the acyl-carbonyl groups and the naphthoquinone framework. This data agrees with the infrared absorption of the acyl-carbonyl groups of these compounds 16–28 that appeared within the range: *ν*_máx_ 1706-1735 cm^−1^.

### 3.2. Voltammetric Measurements and Antioxidant Activities

**Figure 4:**
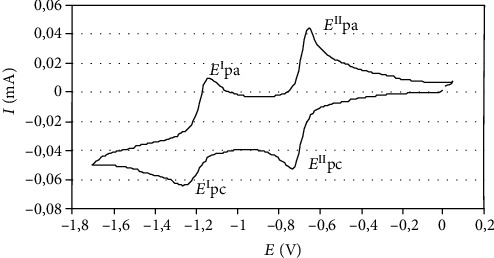
Typical cyclic voltammogram of compound 23 in 0.1 M Et_4_NBF_4_/acetonitrile obtained in Pt electrode, scan rate 100 mV/s. The cathodic (c) and anodic (a) peaks are indicated in the figure.

In order to assess the redox properties of the members of the 2-acyl-3-aminoaryl-1,4-naphthoquinone series 16–28, their half-wave potentials *E*^*I*^_1/2_ and *E*^*II*^_1/2_ were measured by cyclic voltammetry. The measurement was conducted in acetonitrile at room temperature, using a platinum electrode and 0.1 M tetraethylamoniumtetrafluoroborate as the supporting electrolyte [19]. The voltammograms were recorded in the potential range from 0.0 to -2.0 V vs. nonaqueous Ag/Ag^+^.  Figure 4 shows the typical electrochemical behavior of the arylation compound 23 that proceeded in two one-electron diffusion stages.

The cathodic peaks related to the reduction of quinone nucleus, and the anodic one due to its reoxidation, were observed for compound 23 as well-defined quasireversible waves. The *E*_1/2_ values for the first one-electron correspond to the semiquinone radical anion formation and the second one-electron transfer to the dianion formation [17]. The magnitude of these values falls within the ranges −875 to −620 mV/−1295 to −1125 mV for the members of the 2-acyl-3-(4-*N*,*N*-dimethylaminophenyl)naphthoquinones 16-21 and -890 to -685 mV/-1410 to -1180 mV for the members of the 2-acyl-3-(4-amino-2,5-dimethoxyphenyl)naphthoquinones 22-28 (Table 2).

The notable differences of the *E*^*I*^_1/2_ and *E*^*II*^_1/2_ values could be attributed to the stereoelectronic effects of the acyl substituents in the naphthoquinone scaffold (R = methyl, 1-propyl, 1-pentyl, 4-methoxyphenyl, 2,5-dimethoxyphenyl, 2-furyl, and 2-thienyl) in the 4-*N*,*N*-dimethylamino- and 4-amino-2,5-dimethoxyphenyl-1,4-naphthoquinone. Interestingly, the series 22-28 have lower *E*^*I*^_1/2_ values compared to 16-21, with the only exception of 20.

### 3.3. Antitumor Activity

The 2-acyl-3-aminophenylnaphthoquinones 16-28 were evaluated for *in vitro* cytotoxic activities against nontumorigenic human embryonic kidney cells (HEK-293 cells) and two human cancer cell lines DU-145 (prostate) and MCF-7 (mammary) in 72 h drug exposure assays. The cytotoxic activities of the new compounds were measured using conventional microculture tetrazolium reduction assays [29]. Such activities are expressed in terms of IC_50_. Doxorubicin, a well-known anticancer agent currently used in clinical practice, was taken as a positive control. The cytotoxic activity data are summarized in Table 3.

The data indicated that, in general, the DU-145 cells are more sensitive than MCF-7 cells to the compounds. In addition, the 2-acyl-3-(4-amino-2,5-dimethoxyphenyl)naphthoquinone members 22-28 exhibited higher activity than their corresponding analogues 16-21 but lower than those displayed by doxorubicin. Among the 2-acyl-3-(4-dimethylaminophenyl)naphthoquinone members, those containing the furan-2-carbonyl and thiophene-2-carbonyl groups, as compounds 20 and 21, are the most active on the DU-145 cancer cells. Inspection of the biological activity of 2-acyl-3-(4-amino-2,5-dimethoxyphenyl)naphthoquinone members reveals that those containing acetyl and furan-2-carbonyl groups, as 22 and 27, exhibited the higher activity on DU-145 and MCF-7 cancer cell lines. However, by taking the mean IC_50_ value and calculating a selectivity index (IC_50_ HEK-293 value/IC_50_ cancer cell value), compound 22 displays a better index (1.71) than 27 (0.65), suggesting that this latter quinone did not discriminate between cancer and healthy cells. By increasing the number of the aliphatic chain within the acyl substituents, a decrease in the antiproliferative activity is observed: compounds 22 and 23 are more active than 24. The screening also shows that the members having 4-methoxy- and 2,5-dimethoxybenzoyl substituents, as compounds 18, 19, and 25 show weak or almost nonexistent cytotoxic activity. Finally, an additional element may be proposed to explain why compound 22 displays the best activity among the series: namely, its strong electronegative redox potential. In this regard, it is tempting to speculate that compound 22 has higher redox cycling ability, and therefore, it may generate the largest amount of ROS. Indeed, quinone redox cycling has been evoked as the main molecular mechanism explaining numerous deleterious effects by such molecules [43–46].

## 4. Conclusions

We have developed a simple and flexible route for the synthesis of novel 2-acyl-3-(aminophenylamino)-1,4-naphthoquinones whose structure is related to the HTS1, an inhibitor of the heat shock protein Hsp90. The members of the series were prepared in moderate to good yields (63–98%) by reaction of acylnaphthoquinones with phenylamines, catalyzed by CeCl_3_·7H_2_O. The new quinones were characterized by their spectral data and half-wave potentials. The first and second half-wave potential values (*E*_1/2_) of the members of the series are sensitive to the acceptor and donor electronic effects of the substituents located in the quinone double bond. The *in vitro* cytotoxicities of the compounds on cancer cells were determined by using the MTT colorimetric method. The results of the biological evaluation of the 2-acyl-3-(aminophenyl)-1,4-naphthoquinone series show interesting *in vitro* cytotoxic activity on DU-145 and MCF-7 cancer cell lines for the member 22 making it a suitable new chemical entity to be further developed.

## Figures and Tables

**Table 1:** Synthesis of 2-acyl-3-aminophenylnaphthoquinones 16-28 from 2-acylnaphthoquinones 9-15, *N*, *N*-dimethylaniline and 2,5-dimethoxyaniline. 
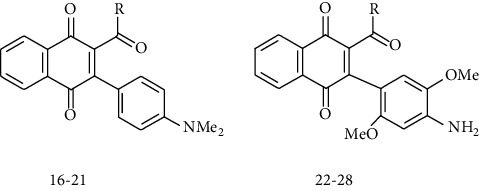

Acylquinone	Product	Structure	Time(hrs)	Yield(%)∗
10	16	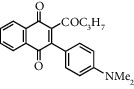	3	65
11	17	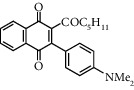	5	33
12	18	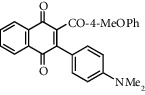	240	91
13	19	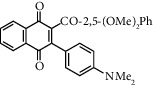	216	81
14	20	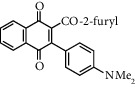	28	98
15	21	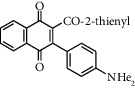	72	94
9	22	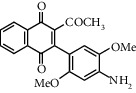	48	77
10	23	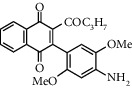	1.5	63
11	24	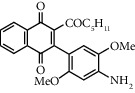	2	63
12	25	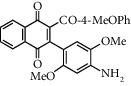	4	91
13	26	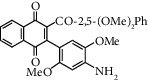	3	87
14	27	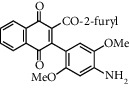	2.5	83
15	28	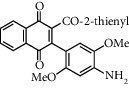	2	91

∗ Isolated by column chromatography. Yields are based on the corresponding acetylnaphthohydroquinones 2-8.

**Table 2:** Half-wave potential values *E*^I^_1/2_ and *E*^II^_1/2_ of 2-acyl-3-aminophenylnaphthoquinones 16-28. 
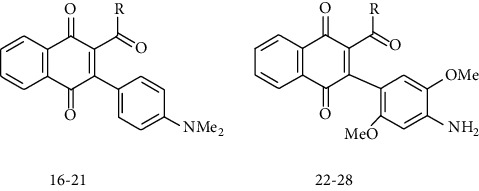

Product no.	R	−*E*^I^_1/2_ (mV)	−*E*^II^_1/2_ (mV)
16	C_3_H_7_	790	1180
17	C_5_H_11_	875	1295
18	4-MeOPh	815	1190
19	2,5-(OMe)_2_Ph	845	1165
20	2-Furyl	620	1125
21	2-Thienyl	775	1165
22	CH_3_	890	1410
23	C_3_H_7_	685	1180
24	C_5_H_11_	755	1230
25	4-MeOPh	720	1225
26	2,5-(OMe)_2_Ph	760	1225
27	2-Furyl	710	1200
28	2-Thienyl	700	1205

**Table 3:** IC_50_ ± SEM (*μ*M) values of 16–28 on DU-145 (prostate cancer cells) and MCF-7 (mammary cancer cells) and nontumorigenic HEK-293 (embryonic kidney cells)^∗^. 
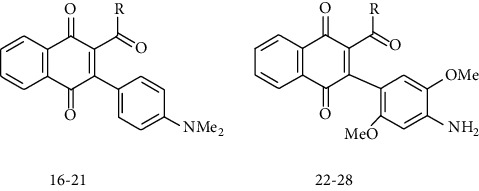

Compound no.	R	DU-145	MCF-7	Mean value	HEK-293
16	C_3_H_7_	77.8 ± 1.1	>100	-	51.1 ± 0.8
17	C_5_H_11_	>100	>100	-	77.4 ± 0.6
18	4-MeOPh	>100	>100	-	>100
19	2,5-(OMe)_2_Ph	>100	>100	-	>100
20	2-Furyl	36.5 ± 0.2	>100	-	35.1 ± 1.4
21	2-Thienyl	53.1 ± 5.0	>100	-	67.6 ± 2.5
22	CH_3_	12.3 ± 0.4	21.2 ± 0.3	16.7	28.6 ± 1.9
23	C_3_H_7_	22.5 ± 0.2	23.3 ± 0.5	22.9	21.5 ± 0.7
24	C_5_H_11_	54.6 ± 1.5	>100	-	58.4 ± 0.6
25	4-MeOPh	>100	73.5 ± 1.6	-	71.3 ± 1.2
26	2,5-(OMe)_2_Ph	28.7 ± 0.7	74.1 ± 2.4	51.4	53.3 ± 1.2
27	2-Furyl	13.2 ± 1.1	21.2 ± 1.4	17.2	11.2 ± 0.7
28	2-Thienyl	24.8 ± 0.3	33.1 ± 1.6	28.9	22.9 ± 0.7
DOX	-	0.70 ± 0.02	0.05 ± 0.003	-	4.27 ± 0.34

^∗^Cells were incubated at 37°C for 48 h, with or without quinone derivatives. Afterwards, aliquots of cell suspensions were taken and the MTT test was performed, as described in Materials and Methods. Results are expressed as mean values ± SEM (*n* = 3). DOX = doxorubicin.

## Data Availability

All data to be shared are included in the main text as well as in Supplementary Materials.

## Supplementary Materials

Supplementary Materials Description of the spectral data of nuclear magnetic resonance (^1^H NMR and ^13^C NMR) and high-resolution mass spectrometry (HRMS) of 2-acyl-3-aminophenylnaphthoquinones.
