# Supplementary material for: New 2-Acetyl-3-aminophenyl-1,4-naphthoquinones: Synthesis and In Vitro Antiproliferative Activities on Breast and Prostate Human Cancer Cells
Source: Oxid Med Cell Longev. 2020 Sep 26;2020:8939716. doi: 10.1155/2020/8939716 (PMC7574025; doi:10.1155/2020/8939716)
Supplement: Supplementary Materials — Description of the spectral data of nuclear magnetic resonance (1H NMR and 13C NMR) and high-resolution mass spectrometry (HRMS) of 2-acyl-3-aminophenylnaphthoquinones. [file 8939716.f1.docx]

**Supplementary Materials**

New 2-acetyl-3-aminophenyl-1,4-naphthoquinones. Synthesis and *in vitro* antiproliferative activities on breast and prostate human cancer cells.

*2-Butiroyl-3-((4-dimethylamino)phenyl)naphthalen-1,4-dione* **16** (65%); purple solid, mp: 125-126°C. IR (KBr) ν_máx_ : 1708, 1662, 1605 and 1262 cm^−1^. ^1^H NMR (CDCl_3_): δ 0.78 (t, 3H, *J* = 7.4 Hz, COCH_2_CH_2_CH_3_), 1.53 (m, 2H, COCH_2_CH_2_CH_3_), 2.28 (t, 2H, *J* = 7.2 Hz, COCH_2_CH_2_CH_3_), 3.03 (s, 6H, NMe_2_), 6.71 (d, 2H, *J* = 9.0 Hz, 3′-H + 5′-H), 7.25 (d, 2H, *J* = 9.0 Hz, 2′-H + 6′-H), 7.76 (m, 1H, 6-H), 7.79 (m, 1H, 7-H), 8.11 (m, 1H, 5-H), 8.16 (m, 1H, 8-H). ^13^C NMR (CDCl_3_): δ 13.6, 16.5, 40.1 (2C), 45.5, 111.4 (2C), 118.0, 126.1, 126.8, 131.8, 131.9 (3C), 133.9, 134.0, 142.7 (2C), 151.5, 183.3, 185.3, 204.1. HRMS (APCI): [M+H]^+^ calcd for C_22_H_21_NO_3_: 347.15214; found 347.15240.

*2-Hexanoyl-3-((4-dimethylamino)phenyl)naphthalen-1,4-dione*, **17** (33%), purple solid, mp: 106-107°C. IR (KBr) ν_máx_: 1706, 1640, 1662 and 1263 cm^−1^. ^1^H NMR (CDCl_3_): δ 0.79 (t, 3H, *J* = 6.9 Hz, COCH_2_CH_2_CH_2_CH_2_CH_3_), 1.11 (m, 4H, COCH_2_CH_2_CH_2_CH_2_CH_3_), 1.49 (m, 2H, COCH_2_CH_2_CH_2_CH_2_CH_3_), 2.30 (t, 2H, *J* = 7.3 Hz, COCH_2_CH_2_CH_2_CH_2_CH_2_CH_3_), 3.03 (s, 6H, NMe_2_), 6.71 (d, 2H*, J* = 9.0 Hz, 3′-H + 5′-H), 7.26 (d, 2H, *J* = 9.0 Hz, 2′-H + 6′-H), 7.76 (m, 1H, 6-H), 7.79 (m, 1H, 7-H), 8.11 (m, 1H, 5-H), 8.16 (m, 1H, 8-H). ^13^C NMR (CDCl_3_): δ 13.9, 22.3, 22.6, 31.0, 40.1 (2C), 43.5, 111.4 (2C), 118.0, 126.1, 126.8 (2C), 131.9 (3C), 133.9, 134.0, 142.7, 142.9, 151.5, 183.3, 186.3, 204.2. HRMS (APCI): [M+H]^+^ calcd for C_24_H_25_NO_3_: 375.18344; found 375.18354.

*2-(4-Methoxybenzoyl)-3-((4-dimethylamino)phenyl)naphthalen-1,4-dione* **18** (91%): purple solid, mp: 211-212°C. IR (KBr) ν_máx_: 1735, 1638, 1598 and 1265 cm^−1^. ^1^H NMR (CDCl_3_): δ 2.94 (s, 6H, NMe_2_), 3.81 (s, 3H, 4″-OMe), 6.56 (d, 2H, *J* = 9.0 Hz, 3′-H + 5′-H), 6.83 (d, 2H, *J* = 9.0 Hz, 3″-H + 5″-H), 7.25 (d, 2H, *J* = 9.0 Hz, 2-H′ + 6′-H), 7.77 (m, 1H, 6-H), 7.82 (m, 3H, 7-H + 2″-H + 6″-H), 8.11 (m, 1H, 5-H), 8.21 (m, 1H, 8-H). ^13^C NMR (CDCl_3_): δ 40.0 (2C), 55.5, 111.2 (2C), 114.0 (2C), 118.5, 126.2, 126.9, 129.3, 131.6 (2C), 131.8 (2C), 131.9, 132.2, 133.9, 134.0, 141.0, 144.4, 151.2, 164.0, 183.8, 186.2, 192.7. HRMS (APCI): [M+H]^+^ calcd for C_26_H_21_NO_4_: 411.14706; found 411.14722.

*2-(2,5-Dimethoxybenzoyl)-3-((4-dimethylamino)phenyl)naphtalen-1,4-dione* **19** (81%), purple solid, mp: 198-199°C. IR (KBr) ν_máx_: 1730, 1643, 1606 and 1264 cm^−1^. ^1^H NMR (CDCl_3_): δ 2.94 (s, 6H, NMe_2_), 3.59 (s, 3H, OMe), 3.79 (s, 3H, OMe), 6.58 (d, 2H, *J* = 9.0 Hz, 3′-H + 5′-H), 6.85 (d, 1H, *J* = 9.0 Hz, 3″-H), 7.08 (dd, 1H, *J* = 9.0; 3.3 Hz, 4″-H), 7.17 (d, 2H, *J* = 9.0 Hz, 2′-H + 6′-H), 7.44 (d, 1H, *J* = 3.3 Hz, 6″-H), 7.76 (m, 1H, 6-H), 7.79 (m, 1H, 7-H), 8.11 (m, 1H, 5-H), 8.20 (m, 1H, 8-H). ^13^C NMR (CDCl_3_): δ 40.1 (2C), 55.8, 56.4, 111.4 (2C), 112.6, 113.8, 118.8, 123.0, 125.8, 126.8, 126.9, 131.5 (2C), 132.0, 132.2, 133.7, 133.8, 141.3, 145.7, 151.0, 153.7, 154.0, 183.8, 186.7, 191.9. HRMS (APCI): [M+H]^+^ calcd for C_27_H_23_NO_5_: 441.15762; found 441.15728.

*2-(Furan-2-carbonyl)-3-((4-dimethylamino)phenyl)naphtalen-1,4-dione* **20** (98%), purple solid, mp; 240-241°C. IR (KBr) ν_máx_: 1737, 1662, 1647 and 1261 cm^−1^. ^1^H NMR (CDCl_3_): δ 2.97 (s, 6H, NMe_2_), 6.43 (dd, 1H, *J* = 3.6, 1.7 Hz, furyl), 6.60 (d, 2H, *J* = 9.0 Hz, 3′-H + 5′-H), 7.07 (d, 1H, *J* = 3.6 Hz, furyl), 7.27 (d, 2H, *J* = 9.0 Hz, 2′-H + 6′-H), 7.49 (dd, 1H, *J* = 1.7, 0.6 Hz, furyl), 7.77 (m, 1H, 6-H), 7.81 (m, 1H, 7-H), 8.13 (m, 1H, 5-H), 8.20 (m, 1H, 8-H). ^13^C NMR (CDCl_3_): δ 40.0 (2C), 111.3 (2C), 112.6, 118.3, 119.4, 126.2, 127.0, 132.0 (3C), 132.1, 133.9, 134.1, 139.6, 145.1, 147.4, 151.3, 152.3, 181.5, 183.2, 185.2. HRMS (APCI): [M+H]^+^ calcd for C_23_H_17_NO_4_: 371.39200; found 371.39222.

*2-(Thiophen-2-carbonyl)-3-((4-dimethylamino)phenyl)naphtalen-1,4-dione,* **21** (94%), purple solid, mp: 219-220°C. IR (KBr) ν_máx_: 1731, 1667, 1643 and 1294 cm^−1^. ^1^H NMR (CDCl_3_): δ 2.96 (s, 6H, NMe_2_), 6.59 (d, 2H, *J* = 9.0 Hz, 3′-H + 5′-H), 6.97 (dd, 1H, *J* = 4.9, 3.9 Hz, thienyl), 7.31 (d, 2H, *J* = 9.0 Hz, 2′-H + 6′-H), 7.48 (dd, 1H, *J* = 3.9, 1.1 Hz, thienyl), 7.59 (dd, 1H, *J* = 4.9, 1.1 Hz, thienyl), 7.78 (m, 1H, 6-H) 7.81 (m, 1H, 7-H), 8.13 (m, 1H, 5-H), 8.21 (m, 1H, 8-H). ^13^C NMR (CDCl_3_): δ 40.0 (2C), 111.3 (2C), 118.3, 126.3, 127.0, 128.2, 131.9, 132.0 (2C), 132.1, 133.9, 134.1, 134.3, 135.1, 140.3, 143.3, 144.4, 151.4, 183.1, 185.2, 186.1. HRMS (APCI): [M+H]+ calcd for C_23_H_17_NO_3_S: 387.09291 found 387.09288.

*2-Acetyl-3-((4-amino-2,5-dimethoxy)phenyl)naphthalen-1,4-dione* **22** (77%), purple solid mp: 190-191°C (lit [25] 208ºC). ^1^H NMR (CDCl_3_): δ 2.20 (s, 3H, COCH_3_), 3.69 (s, 3H, OMe), 3.78 (s, 3H, OMe), 4.07 (br s, 2H, NH_2_), 6.37 (s, 1H, 3’-H), 6.55 (s, 1H, 6’-H), 7.76 (m, 1H, 6-H), 7.79 (m, 1H, 7-H), 8.11 (m, 1H, 5-H), 8.14 (m, 1H, 8-H). ^13^C NMR (CDCl_3_): δ 30.9, 56.1 (2C), 99.1, 108.8, 113.5, 126.2, 126.9, 131.7, 132.2, 133.9, 134.0, 139.5, 140.9, 142.6, 144.9, 151.9, 183.3, 184.0, 200.7. HRMS (APCI): [M+H]^+^ calcd for C_20_H_17_NO_5_: 351.11067 found 351.11105.

*2-Butiroyl-3-((4-amino-2,5-dimethoxy)phenyl)naphthalen-1,4-dione*, **23** (63%), purple solid mp: 145-146°C IR (KBr) ν_máx_: 3465, 3380, 1708, 1665, 1621 and 1273 cm^-1^. ^1^H NMR (CDCl_3_): δ 0.74 (t, 3H, *J*= 7.4 Hz, COCH_2_CH_2_CH_3_), 1.50 (m, 2H, COCH_2_CH_2_CH_3_), 2.37 (m, 2H, COCH_2_CH_2_CH_3_), 3.69 (s, 3H, OMe), 3.76 (s, 3H, OMe), 4.09 (br s, 2H, NH_2_) 6.38 (s, 1H, 3′-H), 6.53 (s, 1H, 6′-H), 7.75 (t, 1H, *J* = 3.7 Hz, 6-H), 7.79 (t, 1H, *J* = 3.7 Hz, 7-H), 8.11 (m, 1H, 5-H), 8.15 (m, 1H, 8-H). ^13^C NMR (CDCl_3_): δ 13.5, 16.3, 45.2, 56.1, 56.3, 99.2, 108.9, 113.5, 126.2, 126.9, 131.7, 132.2, 133.8, 134.0, 139.4, 140.9, 143.1, 145.3, 152.0, 183.5, 183.7, 203.1. HRMS (APCI): [M+H]^+^ calcd for C_22_H_21_NO_5_: 379.14197 found 379.14142.

*2-Hexanoyl-3-((4-amino-2,5-dimethoxy)phenyl)naphthalen-1,4-dione*, **24** (63%), purple solid, mp: 77-78°C. IR (KBr) ν_máx_: 3429, 3377, 1708, 1669, 1620 and 1211 cm^–1^. ^1^H NMR (CDCl_3_): δ 0.81 (t, 3H, *J*= 7.1 Hz, COCH_2_CH_2_CH_2_CH_2_CH_3_), 1.11 (m, 4H, COCH_2_CH_2_CH_2_CH_2_CH_3_), 1.47 (m, 2H, COCH_2_CH_2_CH_2_CH_2_CH_3_), 2.38 (m, 2H, COCH_2_CH_2_CH_2_CH_2_CH_3_), 3.69 (s, 3H, MeO), 3.76 (s, 3H, MeO), 4.07 (br s, 2H, NH_2_), 6.17 (s, 1H, 3′-H), 6.53 (s, 1H, 6′-H), 7.75 (t, 1H, *J* = 3.7 Hz, 6-H), 7.78 (t, 1H, *J* = 3.7 Hz, 7-H), 8.11 (m, 1H, 5-H), 8.15 (m, 1H, 8-H). ^13^C NMR (CDCl_3_): δ 13.9, 22.3, 22.4, 31.0, 43.3, 56.1, 56.3, 99.1, 108.9, 113.5, 126.2, 126.9, 131.7, 132.2, 133.8, 134.0, 139.4, 140.9, 143.1, 145.3, 152.0, 183.5, 183.7, 203.2. HRMS (APCI): [M+H]^+^ calcd for C_24_H_25_NO_5_: 407.17327 found 407.17335.

*2-(4-Methoxybenzoyl)-3-((4-amino-2,5-dimethoxy)phenyl)naphthalen-1,4-dione*, **25** (91%), purple solid, mp: 146-147°C. IR (KBr) ν_máx_: 3453, 3377, 1714, 1671, 1621 and 1212 cm^–1^. ^1^H NMR (CDCl_3_): δ 3.58 (s, 3H, MeO), 3.64 (bs, 3H, MeO), 3.81 (s, 3H, MeO), 3.93 (bs, 2H, NH_2_), 6.20 (s, 1H, 3′-H), 6.54 (s, 1H, 6′-H), 6.82 (d, 2H, *J* = 8.4 Hz, 3″-H + 5″-H), 7.78 (m, 1H, 6-H), 7.21 (m, 3H, 7-H + 2″-H + 6″-H), 8.12 (m, 1H, 5-H), 8.19 (m, 1H, 8-H). ^13^C NMR (CDCl_3_): δ 55.5, 56.1, 56.2, 99.2, 109.7, 113.8 (3C), 126.5 (2C), 127.1, 131.6 (3C), 132.0, 132.6, 133.9, 134.1, 139.2 (2C), 140.8, 152.0, 164.1, 183.7, 183.8, 191.7. HRMS (APCI): [M+H]^+^ calcd for C_26_H_21_NO_6_: 443.13689 found 443.13641.

*2-(2,5-Dimethoxybenzoyl)-3-((4-amino-2,5-dimethoxy)phenyl)naphthalen-1,4-dione*, **26** (87%), purple solid mp:108-109°C. IR (KBr) ν_máx_: 3435, 3377, 1737, 1669, 1621 and 1218 cm^–1^. ^1^H NMR (CDCl_3_): δ 3.45 (s, 3H, MeO), 3.57 (s, 6H, 2xMeO), 3.75 (s, 3H, MeO), 3.93 (bs, 2H, NH_2_), 6.18 (s, 1H, 3′-H), 6.51 (s, 1H, 6′-H), 6.83 (d, 1H, *J* = 9.0 Hz, 3″-H), 7.04 (dd, 1H, *J* = 9.0, 3.1 Hz, 4″-H), 7.29 (m, 1H, 6″-H), 7.78 (m, 2H, 6-H + 7-H), 8.13 (m, 1H, 5-H), 8.19 (m, 1H, 8-H). ^13^C NMR (CDCl_3_): δ 55.9 (3C), 56.5, 99.3, 109.7, 112.5, 113.4, 113.7, 122.7, 126.1, 126.9, 127.0, 132.0, 132.6, 133.7, 133.9, 138.8, 140.8, 152.0, 153.7 (2C), 154.1, 183.7, 184.5, 191.0. HRMS (APCI): [M+H]^+^ calcd for C_27_H_23_NO_7_: 473.14745 found 473.14755.

*2-(Furan-2-carbonyl)-3-((4-amino-2,5-dimethoxy)phenyl)naphthalen-1,4-dione*, **27** (83%), purple solid, mp: 188-189°C. IR (KBr) ν_máx_: 3434, 3387, 1710, 1668, 1621 ad 1214 cm^–1^. ^1^H NMR (CDCl_3_): δ 3.58 (s, 3H, MeO), 3.68 (s, 3H, MeO), 4.00 (bs, 2H, NH_2_), 6.25 (s, 1H, 3′-H), 6.44 (m, 1H, furyl), 6.55 (s, 1H, 6′-H), 7.12 (m, 1H, furyl), 7.54 (s, 1H, 5-H), 7.78 (m, 1H, furyl), 7.80 (t, 1H, *J* = 4.4 Hz, 7-H), 8.13 (m, 1H, 5-H), 8.18 (m, 1H, 8-H). ^13^C NMR (CDCl_3_): δ 56.0, 56.2, 99.2, 109.3, 112.6, 126.4 (2C), 127.1 (2C), 131.9, 132.5, 134.0 (2C), 134.1 (2C), 139.5, 140.9, 146.5, 147.6, 152.1, 180.4, 183.3, 183.7. HRMS (APCI): [M+H]^+^ calcd for C_23_H_17_NO_6_: 403.10559 found 403.10532.

*2-(Tiophen-2-carbonyl)-3-((4-amino-2,5-dimethoxy)phenyl)naphthalen-1,4-dione*, **28** (91%), purple solid, mp: 186-187°C. IR (KBr) ν_máx_: 3447, 3390, 1729, 1668, 1621 and 1214 cm^–1^. ^1^H NMR (CDCl_3_): δ 3.62 (s, 3H, MeO), 3.66 (s, 3H, MeO), 3.97 (bs, 2H, NH_2_), 6.23 (s, 1H, 3′-H), 6.58 (s, 1H, 6′-H), 6.98 (m, 1H, thienyl), 7.55 (dd, 1H, *J* = 3.8, 1.0 Hz, thienyl), 7.61 (dd, 1H, *J* = 4.0, 1.0 Hz, thienyl), 7.78 (m, 1H, 6-H), 7.81 (m, 1H, 7-H), 8.13 (m, 1H, 5-H), 8.19 (m, 1H, 8-H). ^13^C NMR (CDCl_3_): δ 56.1, 56.2, 99.3, 109.6, 113.2, 126.5, 127.1, 128.1 (2C), 131.9, 132.5, 134.0 (2C), 134.2 (2C), 134.5, 135.4, 139.5, 140.9, 152.1, 183.3, 183.6, 185.2. HRMS (APCI): [M+H]^+^ calcd for C_23_H_17_NO_5_S: 419.08274 found 419.08347.
